# Adapt water reserves to climate change: study case Romania

**DOI:** 10.1007/s11356-025-36618-7

**Published:** 2025-06-20

**Authors:** Gabriela Elena Dumitran, Liana Ioana Vuta

**Affiliations:** Hydraulic Machinery and Environmental Engineering Department, National University of Science and Technology Politehnica Bucharest, Spl. Independentei, No. 313, 060041 Bucharest, Romania

**Keywords:** Shallow reservoir, Water footprint, Carbon footprint, Climate change, Floating photovoltaic systems

## Abstract

At the regional level, hydrological factors, catchment properties, as well as the way water reserves are utilized, represent major determining factors of the reactions of lake ecosystems to climate change (CC). In this context, lakes also have a negative impact related to the large amounts of water they can consume through evaporation. This paper quantifies the effects of a small artificial lake—Dridu from Romania (with complex usage) on the environment in the context of CC, estimating the blue water footprint (WF) and carbon footprint (CF) under different use scenarios. Thus, an analysis of the evolution of CF and WF is conducted until 2100, considering forecasts of changes in average temperatures, as well as a partial coverage of the lake’s surface with floating photovoltaic systems (FPV). During the study period from 2017 to 2021, the average WF was 0.054 million m^3^, and the carbon intensity was 120.23 kg CO_2_e/MWh. By covering 2 ha of the reservoir’s surface with FPV, the production and release of CO₂ and CH₄ into the atmosphere decrease, on average, by 30% for WF and 28.13% for CF. For the CC scenarios, it is observed that WF will increase by an average of 10.4%, while the carbon footprint shows no significant variations. It is worth mentioning that this type of approach, e.g., impact of FPV on WF and CF, as well as the use of FPV as a measure of coping with CC, has not been studied yet in any region of Eastern Europe. This study, given that it deals with relatively new technologies (FPV), has some gaps, since the CF is computed based on estimates, not on measured data. However, the results and the methodology currently presented can be used by various stakeholders to identify the best coping mechanism for preserving the water quantity and for generating clean energy. Also, these types of studies can be a good stimulus for the authorities to develop and permit the placement of FPV on small reservoirs and to investigate the effects of such projects in terms of energy, FPV efficiency, water quantity, and quality, as it will allow the actual quantification of different effects and benefits brought by this technology (water quality indicators, GHG emission, reduction of evaporation, FPV energy output).

## Introduction

Implementing low-carbon renewable energy technology is essential in ensuring *access to affordable, reliable, sustainable, and modern energy for all* (IEA 2019), as outlined in the United Nations Sustainable Development Goal (SDG) No. 7 (https://sdgs.un.org/goals), which was adopted by UN member states in 2015 (United Nations 2015). Many studies have emphasized the advantages of clean energy as a reliable, long-term energy source. Renewable energy development and use require careful planning to achieve the economic, environmental, and social goals of sustainable development (Mostafaeipour and Le 2024). However, in humanity’s attempts to find quick solutions for the climate problem, it is important to ensure that new energy developments do not undermine other SDGs (Venturini et al. 2024).

Hydropower (HP) is a mature technology and, although it is considered a low-carbon energy source, greenhouse gas (GHG) emissions from reservoirs can lead some hydroelectric plants to have high GHG intensity (emissions per unit of electricity generated). Almeida et al. underline the global potential for offsetting GHG emissions from HP by installation of FPV on existing reservoirs, and estimate that these actions could allow, in 2024, for more than half of the world’s high GHG-emitting hydropower plants to reach low GHG-emission energy targets and also support larger-scale climate change mitigation efforts (Almeida et al. 2024). Photovoltaic panels are an ideal fit on the surface of water reservoirs in HP facilities (Vidovic et al. 2023). Thus, integrating FPV systems with the existing hydroelectric power plants (HPP) would increase renewable energy (RE) production (Berga 2016), without taking up valuable land, and additionally, would control the evaporation phenomenon at the surface level of the reservoirs (Jin et al. 2023), retaining water for hydropower, drinking, and irrigation (Victor et al. 2014). Despite all of this, many aspects remain largely unstudied due to the novelty of the concept and the early stages of expansion and development (Karipoğlu et al. 2024). Because the HP reservoirs already have the grid infrastructure for conveying electricity to consumers, the transmission costs will be reduced. In this way, we can reduce the CF and WF for HPP, on the one hand by obtaining a quantity of energy based on a technology that has a median carbon intensity (Li and He 2022), and on the other hand by reducing evaporation from the lakes by covering part of the water surfaces with FPV. It is using the concept of the WF as an indicator of hydroelectricity’s water consumption, and in this report, the analysis is limited to the quantification of the blue water footprint of HPP, which focuses on the evaporation processes of water from the HP reservoirs (Mekonnen and Hoekstra 2012). On the other hand, the CF of HPP is defined as the sum of GHG emissions expressed as CO_2_ equivalents due to dam construction, reservoir impoundment, and HP production (Li et al. 2019).

Nowadays, the deployment of FPV power plants appears to be a viable alternative for sustaining a more significant development in energy generation, with the ability to use vast water surfaces being very important (Xiong et al. 2023). Increasingly, floating photovoltaic solar installations are emerging as promising renewable energy solutions worldwide (Ramanan et al. 2024), and in this regard, it is estimated that FPV could generate 9434 TWh/year if installed on approximately 30% of the available reservoir surface area (Jin et al. 2023). Similarly, Sanchez in 2021 estimates that in Europe, FPV has the potential to double energy generation (Sanchez et al. 2021), while water evaporation can be reduced by approximately 557,000 l in the EU (Kakoulaki et al. 2023). Globally, FPV technology is experiencing rapid growth, so by the end of 2023, Nobre identified 650 operational installations, of which 66.2% are installed on lakes with an area smaller than 0.1 km^2^ and with average coverage of 34.2% (35% in Asia and 28% in Europe and North America) (Nobre et al. 2024).

The technical benefits of FPVs are well recognized, but their impact on the environment is less understood (Gorjian et al. 2021; Benjamins et al. 2024), whether it is positive or negative. This impact is felt on the physical, chemical, and biological processes in the water body and is strongly influenced by the design of the FPV, the properties of the water body, the location of deployment, and the climatic conditions (Armstrong et al. 2020). However, Nobre et al.’s results indicate that the potential ecological effects of FPV on water bodies are expected to be primarily determined by the level of FPV coverage, and in this regard, smaller lakes are more likely to have higher FPV coverage and therefore a more intense potential ecological impact, mainly caused by high levels of light penetration reduction and changes in water temperature (Nobre et al. 2024). Also, according to Li, the main mechanism of the impact of FPV on the ecological environment is to disrupt the original radiant energy balance of the installation area, and then act on factors such as temperature, wind speed, turbulence, and precipitation (Li et al. 2022).

HP contributes to CC mitigation through the crucial role it can play in the decarbonization of energy production. HP and CC have a dual connection: on one side, HP is a renewable energy (RE) source that helps reduce greenhouse gas (GHG) emissions and mitigate global warming; on the other hand, CC is expected to alter river discharge, affecting water availability and HP generation. The consequences of CC can be significantly mitigated by adaptation, which entails taking actions to reduce vulnerability to climate change on a continuous basis (Masood et al. 2023). The water resources of lakes and reservoirs serve as particularly effective indicators for the current CC for several reasons: they are globally distributed, covering a wide range of geographic locations and climatic regions; they are well-defined ecosystems that have been studied extensively; they respond directly to climate change and incorporate its effects within the catchment; and they integrate responses over time (Adrian et al. 2016). For example, O’Reilly in 2015, in an extensive global study based on infrared thermal images from satellites of lake surface temperatures during the summer, found an average trend of lake surface warming of 0.34 °C for the period 1985–2009 (O’Reilly et al. 2015). Global warming’s impact on precipitation and drought is projected to worsen the issue of water scarcity, and to mitigate the impact of water shortages caused by precipitation variability, it is estimated that the global water storage capacity is around 8070 km^3^ (Lehner et al. 2011). For shallow reservoirs, the estimated total evaporating surface area exceeds 300,000 km^2^ (Lehmann et al. 2019), and evaporative losses from such surfaces may correspond to a significant reduction of storage (even 50% in reservoirs in arid regions) (Craig 2005). Since shallow reservoirs (depth < 20 m) are associated with high carbon intensity (Li et al. 2022), and the most significant benefit of FPV is a 70–80% reduction in evaporation losses (Lehmann et al. 2019; Redón Santafé et al. 2014; Scavo et al. 2021; Santos et al. 2022; Ilgen et al. 2024), covering part of the lake’s surface with FPV could represent a solution for improving the trophic status of the reservoirs.

Rahaman’s 2025 analysis, based on the floating equivalence ratio, studies the multiple advantages of FPV systems, demonstrating their viability as a sustainable energy alternative within the water–energy nexus. Therefore, the analysis indicates a substantial decrease in evaporation, negligible effects on aquatic ecosystems, and long-term advantages when compared to the ground-mounted PV system, such as total water savings of 80% and total CO_2_ savings of 15.31% over a 30-year operational timeframe (Rahaman et al. 2025*).* Tama and Vicente in 2023 use life cycle analysis in their study to assess the magnitude of the environmental impact, in terms of global warming potential and water footprint, over the 20-year useful life of a rural electrical energy concession made up of 120 Wp household photovoltaic systems in isolated communities in the Peruvian Amazon region, and report a reduction of carbon footprint by 64.5% and water footprint by 95.84% (Tama and Vicente 2023). The study by Razeghi-Jahromi rigorously analyzed the positive and negative aspects of floating photovoltaic systems relative to land-based installations using content analysis, concluding that floating photovoltaic systems demonstrate an 18.18% higher efficacy in reducing greenhouse gas emissions compared to land-based systems (﻿Razeghi-Jahromi et al. 2024).

The ideas of carbon footprint and water footprint were first proposed in the early 2000 s as methods for monitoring greenhouse gas emissions and water use in connection to consumption patterns (Ercin and Hoekstra 2012).

At this point, the literature review does not reveal any study dealing with the estimation of WF and CF related to FPV; thus, a direct correlation between these two footprints and FPV is lacking. The main directions of the research when referring to FPV are focused either on electricity generation efficiency compared to the ground-mounted photovoltaic panels or on the influence of FPV on water quality. The latter direction is also not very well documented, since there are just a few reservoirs that are currently monitored in terms of water quality indicators; thus, a generally accepted theory is still missing. Usually, the literature reports the effects of FPV on the evaporation rate and the effect of water on the FPV’s temperature, e.g., the higher efficiency of FPV compared to ground PV systems.

The present study aims to assess the change of WF and CF for a shallow Romanian reservoir—Dridu (with hydropower use)—when covering a percentage of the surface of the reservoir with FPV.

Based on the values of weather variables in the area of the Dridu reservoir (Zippenfenig 2023), as well as the energy delivered by the micro hydroelectric power plant (MHPP) Dridu between 2017 and 2021, the blue WF related to evaporation using only gross water consumption and CF were computed. On the other hand, for investigating the future trends in WF and CF, Climate Information Portal has been used to obtain the climate change prediction coefficient for RCP 4.5 scenarios (https://climateinformation.org). Furthermore, this paper analyzes how the blue WF and CF are modified if 2 ha of Dridu reservoir’s surface is covered by FPV, and also for the future CC scenario. Additionally, given the trophic state of Dridu reservoir, which is a shallow reservoir located in a plain area, very vulnerable from the point of view of eutrophication, partially covering the surface of the reservoir with FPV is expected to reduce this phenomenon.

## Literature review

Climate change will affect water availability in Europe and will put more stress on water resources and water-related sectors of the economy. According to the Intergovernmental Panel on Climate Change, rising stream temperatures and nitrogen loading in rivers are two ways that climate change will affect water quality (Portner et al. 2022). The impact of climate change on water resources is complex and varies by location; however, substantial evidence indicates that climate change is exacerbating stress on already vulnerable water sources (Skaland and Wong 2022).

Several studies have found that climate change has a significant impact on the water sector (Papadimitriou et al. 2019). Thus, Elgendy et al. (2024) suggest that rising temperatures and evapotranspiration rates can lead to increased water demand, while variations in precipitation patterns, including intensity (Elgendy et al. 2024) and intervals between occurrences (Garnier and Holman 2019), may occur. Increased precipitation intensity may worsen floods, affecting human lives and property (Yang et al. 2014), but extended dry periods and low precipitation volumes may cause droughts, compromising water and food security. Higher temperatures and changes in precipitation patterns exacerbate water scarcity, intensify the occurrence and severity of droughts and floods, and facilitate the mineralization of nitrogen, phosphorus, and carbon from soil organic matter. Water contamination can lead to an overabundance of inorganic nutrients in ecosystems, driving eutrophication (Galindo-Miranda et al. 2019), limiting water use for different purposes. Simultaneously, higher rainfall intensity results in increased surface runoff and soil erosion, hence exacerbating the transport of inorganic fertilizers and other pollutants to surface rivers and lakes (Ramião et al. 2024).

Climate change impacts river flow by altering precipitation patterns, timing, frequency, and duration, among other meteorological variables. Human activities, including deforestation and fossil fuel combustion, have contributed to rising global surface temperatures, making climate change a major environmental concern (Zhao et al. 2023). On the other side, human activities such as land use, dam exploitation, and water intake have a significant impact on river flow and timing (Langhammer and Bernsteinová 2025). Didovets et al. in 2020 attempted to produce a general pattern of climate change impacts on river discharge in Ukraine and analyze it. Their results show that river discharge is expected to decrease in most of the Ukrainian river basins in the middle and end of the twenty-first century; in particular, a shift of the spring river discharge peak to earlier months is projected, and also in summer months, river discharge would decrease in most basins (Didovets et al. 2020).

Intentional and planned responses to identified impacts and vulnerabilities are necessary for effective climate change adaptation. According to the EEA report Europe’s State of Water 2024, adaptation is essential to guaranteeing water supplies because climate change is predicted to increase the demand for irrigation water, putting more strain on Europe’s water resources (EEA 2024). Reducing water use, establishing efficiency goals, enhancing management techniques, and utilizing nature-based solutions (restoring wetlands, floodplains, and rivers) are all necessary to adapt to climate change. To reduce the harm caused by the shifting effects of climatic factors, development methods must adapt human or natural systems (Wardropper and Rissman 2019). Among these measures are reactions to regional hydrology and ecology, as well as actions that help build climate change resistance. Many researchers concur that an integration of climate change adaptation strategies is required, as no single solution can effectively handle all future concerns (Bartletta and Dedekorkut-Howes 2022).

Wojkowski et al. in 2023 analyze the potential loss of water storage capacity due to climate change in selected river basins in Central and Eastern Europe, using the nondimensional indicator—landscape water potential. It thus calculates the climatic water balance as the difference between annual precipitation and annual potential annual evapotranspiration for two-time horizons (2021–2050) and (2050–2100), considering the climate scenarios RCP 4.5 and RCP 8.5. This study demonstrated the interconnection and potential synergistic impacts of climate change and land use and land cover conversion, elucidating the total impact of environmental changes on runoff. The authors suggest that effective land use and land cover management can positively influence water storage in watersheds, reflected in an increase of the landscape’s water potential, and may contribute to reducing the adverse impacts of climate change (Wojkowski et al. 2023).

In Romania, as elsewhere worldwide, there is a growing need to understand the hydrological response to climate change, particularly in considering the anticipated decline in mean streamflow and the increasing hydrological droughts affecting water consumers in this region (Van Vliet et al. 2015). It has already been shown that global warming has an impact on the seasonality of streamflow in Romania (Birsan et al. 2014; Zaharia et al. 2019), and decreasing trends in streamflow variability have been observed for rivers in southern Romania (Birsan 2015; Zaharia et al. 2016). The southern region is the most vulnerable area at CC in Romania, with increasing aridity, climatic water deficit (Prăvălie et al. 2019), and a ratio between evapotranspiration to precipitation with higher values than in other sub-basins of the Danube (Malagò et al. 2018).

Danielescu et al. in 2022 analyzed the impact of future climate change on hydrological processes in a Romanian watershed in the short (2021–2050) and long term (2071–2100). They estimate that precipitation will decrease by up to 7.5% and temperature will increase by up to 4.2 °C, while in the long term there will be a decrease in soil water content (between 14 and 21.5%) and streamflow (between 4.2% in RCP 4.5 and 9.7% in RCP 8.5). Also, snowpack will be reduced by more than 40% in the long term, which will have an impact on the temporal dynamics of streamflow (Danielescu et al. 2022).

To limit the effects of climate change on water resources, proactive, urgent, and sustainable adaptation efforts are required, which, according to Victor et al. 2014, refer to processes of adjustment to the current or projected climate and its impacts (Victor et al. 2014). Thus, according to their goals, these directions can be divided into two categories: (1) lowering GHG emissions and (2) adapting systems to expected future CC (Saraswat et al. 2016).

In a review of 131 important studies on CC adaptation solutions in water management published in the last two decades, Elgendy et al. in 2024 differentiate these strategies according to whether they use or not hydrological, hydraulic, and water management models (Elgendy et al. 2024). Thus, the CC adaptation strategies without models may be founded on: 1) governmental policies that promote climate change adaptation in water management, particularly for transboundary basins; 2) enhancing urban resilience primarily against flood risks by employing methodologies centered on intelligent water utilities, collaborative programming of diverse water sources, and integrating water system management with flood control measures utilizing artificial intelligence, permeable pavements, rainwater harvesting systems, and bioretention; 3) locally implemented actions through simple methods that empower communities to manage increased drought, utilizing canals; and 4) ecosystem-based approaches with measures at the smaller scale like drains, contour walls, and home gardens, or for the watershed-scale like a) afforestation or reforestation, which lowers greenhouse gas emissions and reduces flooding by increasing stormwater infiltration and capture; b) riparian vegetation strips near watercourses to regulate water quality and quantity and minimize soil erosion; c) using natural wetlands to promote groundwater recharge, reduce flood peaks, and improve stormwater quality in urban settings; 4) techniques based on mangroves, coastal marshes, dunes, and barrier reefs which reduce the impact of storm surges on hilly areas and prevent structural devastation by absorbing wave energy (Khaniya et al. 2021).

Also, CC adaptation strategies that have incorporated modeling can also be considered, which are divided into two categories according to their goals: flood mitigation and storm drainage strategies, and water resource management strategies. While surface water drainage and flood mitigation strategies aim to reduce surface runoff or update master plans, water resource management strategies aim to minimize the future water supply–demand gap, maintain the water resource allocation scheme, or optimize it to maximize net revenue net benefits under CC. These final measures can be divided into three groups: (1) water supply conservation, water demand management, and water reuse adaptation strategies; (2) reservoir management and reservoir control curve updates; (3) water allocation adaptation through various methods.

Wojkowski et al. (2023) underscore the significance of strategies focused on mitigating climate change through the reduction of greenhouse gas emissions; however, the absence of sustainable land management policies may undermine the beneficial impact of decreased carbon dioxide or methane emissions on the environment (Wojkowski et al. 2023).

In 2014, Ludwig et al. examined the main similarities and distinctions between climate change adaptation and integrated water resources management, considering the significant uncertainty in future forecasts of climate change consequences. They have therefore created two totally different approaches to adaptation in response to these uncertainties: one bottom-up approach, which essentially ignores uncertainty and concentrates on reducing vulnerabilities by creating resilient water systems, and one top-down approach, which is based on extensive biophysical impact analyses and focuses on quantifying and minimizing uncertainty by using a range of scenarios and various climate and impact models. According to the study’s findings, neither strategy should be used alone in water management because the top-down strategy creates a lot of uncertainty and makes decision-making more difficult, while the bottom-up strategy concentrates too much on socioeconomic vulnerability and not enough on creating technical solutions. Therefore, the researchers propose that risk-based adaptation to CC is a more promising approach (Ludwig et al. 2014).

In Kumar et al.’s 2016 study, for each future climate change scenario, a detailed planning of scenarios for regional water demand and supply is conducted for three major types of users: domestic, industrial, and agricultural. The analytical method used is based on hierarchical models for decision support with hierarchical structures of criteria and on the prioritization of alternatives, thus obtaining a classification of a set of possible actions in terms of different types of indicators, such as costs, water stress, and environmental impact. In this regard, various climate change adaptation measures are compared, and the sustainability of management actions is tested for different climate change scenarios, considering measures such as water transfer between basins, sectoral demand management from industry, agriculture, and the domestic sector, as well as alternative water sources like recycled and desalinated water. The results obtained showed that the use of alternative water resources is the most reliable alternative, with an average reuse of recovered water in industry and agriculture and a low to medium use of desalinated water in the domestic and industrial sectors (Kumar et al. 2016).

With regard to water reserve adaptation, it can be mentioned the European Union Mission on Adaptation to CC, which aims to empower EU regions, cities, and local authorities to become more resilient to CC.

Ramião et al., in their study, indicate that climate change will increase the necessity for deliberating and implementing ecological flows due to reduced river discharge and vulnerability to water abstraction. The strategy to protect Europe’s water resources has previously emphasized the critical need to better handle over-abstraction of water, the second most common pressure on EU ecological status (European Commission 2015), which can be exacerbated by climate change. As a result, it is critical to develop adaptation measures that maintain enough water supply while preserving freshwater ecosystems and the benefits they provide (Ramião et al. 2024).

For better adaptation to CC, numerous cities have started to use various strategies like collecting rainwater to reduce the consumption from the centralized distribution system and to diminish the stress on the stormwater collection system, reforestation of areas prone to flash floods for diminishing the surface runoff and for favouring the infiltration and groundwater supply (Brotto et al. 2019).

Other actions pursue restoring wetlands and reconnecting the rivers to natural floodplains, which can lead to increased flood resilience, greater biodiversity, and support for local economies. Such an example is the Lower Danube Green Corridor Agreement, which began in 2000, promoted by Bulgaria, Romania, Ukraine, and Moldova, and the estimated cost is 183 million euros. Thus, several flood-protecting dikes have been removed, allowing the river to freely flow on its natural course, and reforestation actions have been performed. The extensive benefits of restoration encompass flood and drought management by water retention and gradual release, groundwater renewal, and water purification through filtration, natural resource production (e.g., fish and reeds), and recreational activities (Hulea et al. 2009).

## Conceptual framework

As stated before, the objective of this work is to assess the diminish of WF and CF of Dridu reservoir, when, beside the current uses (hydropower, flood control, etc.), it is combined with a FPV system. In order to achieve accomplish that, the following methodology has been used.

First, it was assessed the blue water footprint for the 5 years between 2017 and 2021, by considering a 10% of the reservoir services corresponding to hydropower. WF of HPPs is 99% due to evaporation (Wang et al. 2019), which can be calculated with the Penman–Monteith equation. This equation requires meteorological data: shortwave and longwave radiation, wind speed, air temperature, relative humidity, and precipitation.

Second, the carbon footprint has been computed, based on the equations proposed by Scherer. For applying these equations, data regarding area-to-electricity ratio and reservoir surface are needed for assessing the CO_2_ emissions, and age of functioning and maximum temperature must be known for assessing the CH_4_ emissions. The total CF is given by the sum of the two components, thus CH_4_ emissions must be converted to carbon equivalent emissions.

Third, the FPV system has been investigated using Global Solar Atlas software and PVSyst V7.4.7. Each software requires data regarding the location of the studied system (latitude and longitude), and details regarding the intended installed power. As results, it provides details regarding the total photovoltaic power output, horizon and sunpath, monthly average of the photovoltaic power output, etc. Afterwards, the WF and CF were recomputed, considering a part of the reservoir surface covered by the FPV system.

Fourthly, the CC has been assessed based on data reported on Climate Information Portal (https://climateinformation.org), and, knowing the prediction regarding the temperature in the Dridu reservoir area, new values of WF and CF have been obtained.

Finally, the decrease in CO_2_ emissions has been estimated, by considering the energy generated by the FPV system and applying the conversion factor reported by Nowtricity.

## Materials and methods

### Study area

Romania is characterized by a temperate-continental climate and, according to numerous studies, the southern part of the country has been facing significant water stress for a considerable period of time, and numerous changes in the availability of water resources have been reported. The national report on State of the climate, 2024, presents an increase in the average annual temperature by 2.11 °C, which is 0.30 °C above the average global temperature and 0.12 °C greater than the average temperature in Europe (1.99 °C) (Antonescu et al. 2024).

Thus, Romania is facing numerous challenges brought by climate change: increasing temperature, more frequent and intense heat waves, longer and repeated droughts. However, if many regions over the country cannot be characterized by a decisive diminishing trend in water resources (some of the regions being expected to have higher precipitations and water flows), the southern part is definitively under stress. The same national report forecasts that, in RCP 8.5 scenario, the average annual temperature in Romania will increase by 4 °C by 2100 and the southern and southeastern areas will experience heatwaves prolonged up to 5 months/year.

Dridu reservoir is located on Ialomita River and is in operation from 1988. For this basin, several studies report changes in the water resources over time. In 2022, Chelu A. et al. conducted a study on four hydrographic basins in the southern part of the country, including the Ialomița River basin, in which Dridu reservoir is located. The aim was to assess the main drivers between climate variability and anthropogenic activities which impacted the river water resources. Thus, based on data from 1966 to 2010, it was concluded that streamflow decrease in Ialomița hydrographic basin is mainly due to climate (79.2%) (Chelu et al. 2022). These findings are in agreement with previous studies which have been conducted on the same hydrographic basins (Retegan and Borcan 2011; Borcan et al. 2014).

Dridu reservoir is a shallow one (Fig. 1) with total surface area 1040 ha, total volume 20.16 M m^3^ and maximum depth 11 m, which fulfils the following purposes: a) mitigation of floods on Ialomita River; b) industrial water supply with a flow of 1.5 m^3^/s; c) irrigation of 17,000 ha, with a flow of 20–50 m^3^/s; d) production of electricity by MHPP with an installed power of 2.4 MW and an estimated annual production of 4.76 MWh/y, for a flow of 24 m^3^/s.

**Fig. 1:**
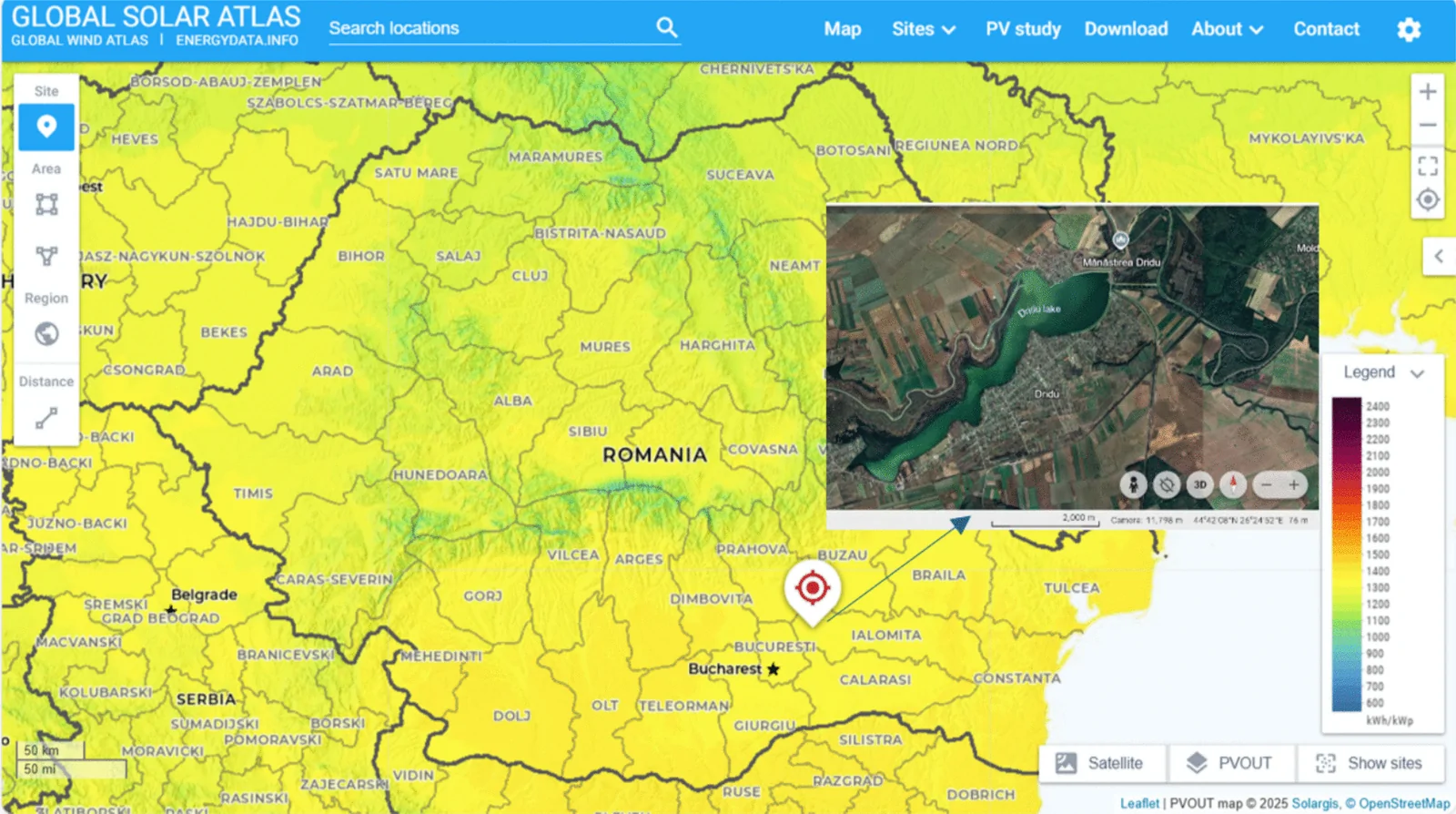
Map of Romania showing the specific photovoltaic power output and Ialomiţa catchment/Dridu reservoir

The power plant is equipped with four EOS horizontal axis turbines, directly coupled with the generators, producing 4.76 MWh in a normal hydrological year with an average operating time of 2125 h.

### Blue water footprint

WF for reservoirs response methods are still being investigated, while CF reduction and balancing techniques for aquatic ecosystems have already been created and put into practice (Ercin and Hoekstra 2012). Considering the uses of Dridu development, in this study a total WF allocation was made considering ecosystem services, setting the values of the allocation coefficient as follows: 60% for flood control, 15% for irrigation and industrial water supply, and 10% for hydropower. The WF of electricity generated by Dridu MHPP is calculated for the 2017–2021 period by dividing WF of the reservoir to annual energy production.

The whole life cycle water footprint of hydropower plants consists of evaporation (greater than 99% of total WF), construction stage, and operation stage (< 0.1% of total WF) (Wang et al. 2019). In this study, only the WF of evaporation was considered, which can be estimated according to meteorological data with the following formula:

1 $$\mathrm{WF}=k\bullet \left(10\times \sum\nolimits_{0}^{365}E\right)x\bullet A [{\mathrm{m}}^{3}/\mathrm{year}]$$

where *E* is daily evaporation [mm/day]; *A* is area of the reservoir [ha]; *k* is the area correction factor (*k* = 0.5625), which considers that the reservoir surface at average filling conditions is smaller than the maximum area reported; 10 is a constant used to convert the measurement units (Hogeboom et al. 2018). The evaporation from the water surface is estimated with the Penman–Monteith (Mekonnen and Hoekstra 2012) equation. As a result, the weather information downloaded from the meteorological site open-meteo.com (Zippenfenig 2023), which offers meteorological and solar data for the Dridu area, is used as input data. Datasets for the meteorological variables of shortwave and longwave radiation, wind speed, air temperature (Fig. 2), relative humidity, and precipitation for the five years studied were used to estimate the evaporation and, after that, to compute the WF. In Fig. 2, the month number 1 represents January of the year 2017—the start date of the study period—while the month with the number 60 represents December of 2021.

**Fig. 2:**
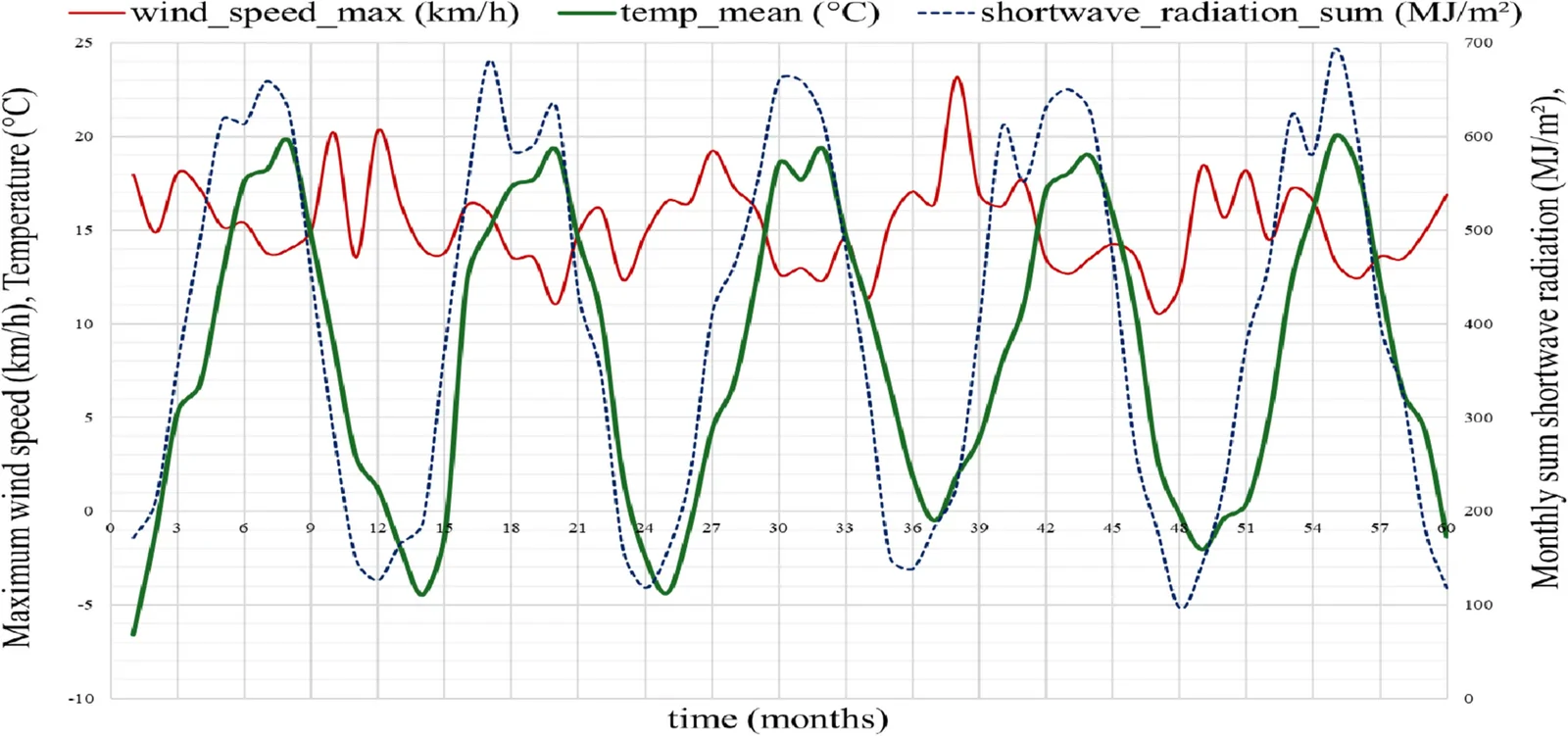
The mean temperature, wind speed and shortwave radiation in Dridu area

### Carbon footprint

Carbon emissions per energy unit expressed in [kg/MWh] were estimated using the equations proposed by Scherer, as follows:

2 $${\mathrm{CO}}_{2}=-169.73+241.86\bullet \mathrm{AE}+120.34\bullet \mathrm{ln}\left(A\right), [\mathrm{kg} \mathrm{CO}2/\mathrm{MWh}]$$

 

3 $$\mathrm{ln}\left({\mathrm{CH}}_4\right)=-9.81-0.75\bullet\mathrm{ln}\left(\mathrm{AGE}\right)+1.18\bullet\mathrm{ln}\left(\mathrm{AE}\right)+4.5\bullet\mathrm{ln}\left(T_\mathrm{max}\right),\lbrack kg\;CH_4/MWh\rbrack$$

where the CO_2_ emissions per energy unit depend on the area-to-electricity ratio (AE) in [km^2^/GWh] and reservoir surface (*A*) in [km^2^], while the CH_4_ emissions are based on the age of functioning (AGE) expressed in [years], and maximum temperature (*T*_max_) in [°C]. CF was calculated as the sum of the two emissions, obviously after transforming CH_4_ emissions to carbon equivalent emissions (Scherer and Pfister 2016).

### FPV simulation and CC scenario

For Dridu reservoir, a FPV plant with a capacity of 3 MW was designed, and the simulation was performed using two software: Global Solar Atlas 2.0 (https://globalsolaratlas.info/map) and PVSyst V7.4.7 (https://www.pvsyst.com). Data for CC scenarios are split into three intervals: first between 2011 and 2040, second from 2041 to 2070, and third from 2071 to 2100, reporting for the Dridu catchment increases in air temperatures of 1.33 °C; 2.4 °C; respectively 3.44 °C for the three analysis periods (Zhou et al. 2021).

## Results

Using the two mentioned models for Dridu location, the annual global horizontal irradiation (GHI = 1.376 MWh/m^2^) and the plane-of-array irradiation (POA = 1.6 MWh/m^2^) were calculated. The PV panels are placed at an optimum tilt angle from the horizontal surface, which is 10°, while the albedo coefficient, a measure of the irradiance reflected by the PV panel, was 0.2. In the preliminary design of the model FPV system, the major factors responsible for the maximum output power are the direction and position of the FPV array, and in this respect, the modules should be placed in south direction with the optimum tilt angle (*β* = 10°) of the PV panels and an azimuth angle equal to 180°. The installation of a 3 MW FPV plant at Dridu reservoir will result in a power density of 150 MW/km^2^, an annual energy of 3.253 GWh/year, an energy density of 162.65 GWh/km^2^, a performance ratio of 67.77%, and a specific yield of 1084 kWh/kWp. Based on preliminary designs of FPV systems for Dridu reservoir, the average area required for a 3 MW FPV plant was determined to be almost 2 ha, resulting in land savings of 0.2 km^2^. The maximum monthly evaporation rate for Dridu reservoir, of 170 mm, was observed during July 2021, while the minimum one, 9 mm, was registered in December 2020 (Fig. 3).

**Fig. 3:**
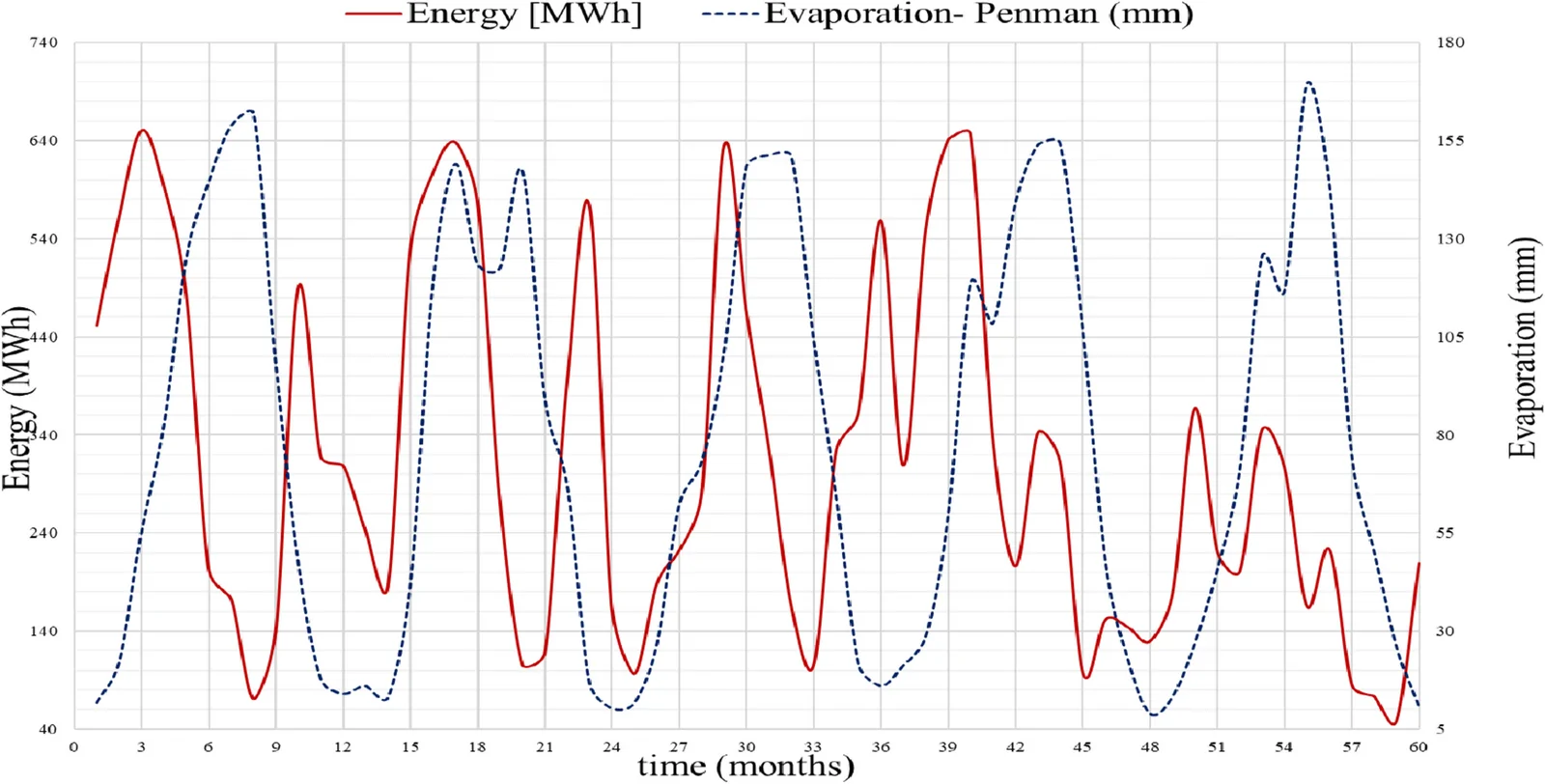
The evaporation in Dridu area and energy for MHPP Dridu

Based on the values of the energies delivered in the system by Dridu MHPP and the weather variables in the area of the reservoir, the WF for the 5 years analyzed was determined, which has a mean value of 0.054 10^6^ m^3^(Fig. 4). Also, the quantity of water conserved, both directly and indirectly, was computed based on the proposed methodology by Ravichandran et al. (2022). The results show that, based on the evaporation rate at the Dridu location, installing an FPV plant will save directly 0.017 10^6^ m^3^/year of water. Additionally, based on the equation proposed by Liu et al. (2017), for the Dridu reservoir, an indirect water saving of 0.072 10^6^ m^3^/year of water is obtained with a very small area ratio (0.19%), which shows that placing this system on the water surface will not affect the ecosystem. A value of 0.089 10^6^ m^3^/year for water savings is well correlated with the values obtained by Hayibo in 2022 or Razeghi-Jahromi in 2024 (Hayibo and Pearce 2022; Razeghi-Jahromi et al. 2024).

**Fig. 4:**
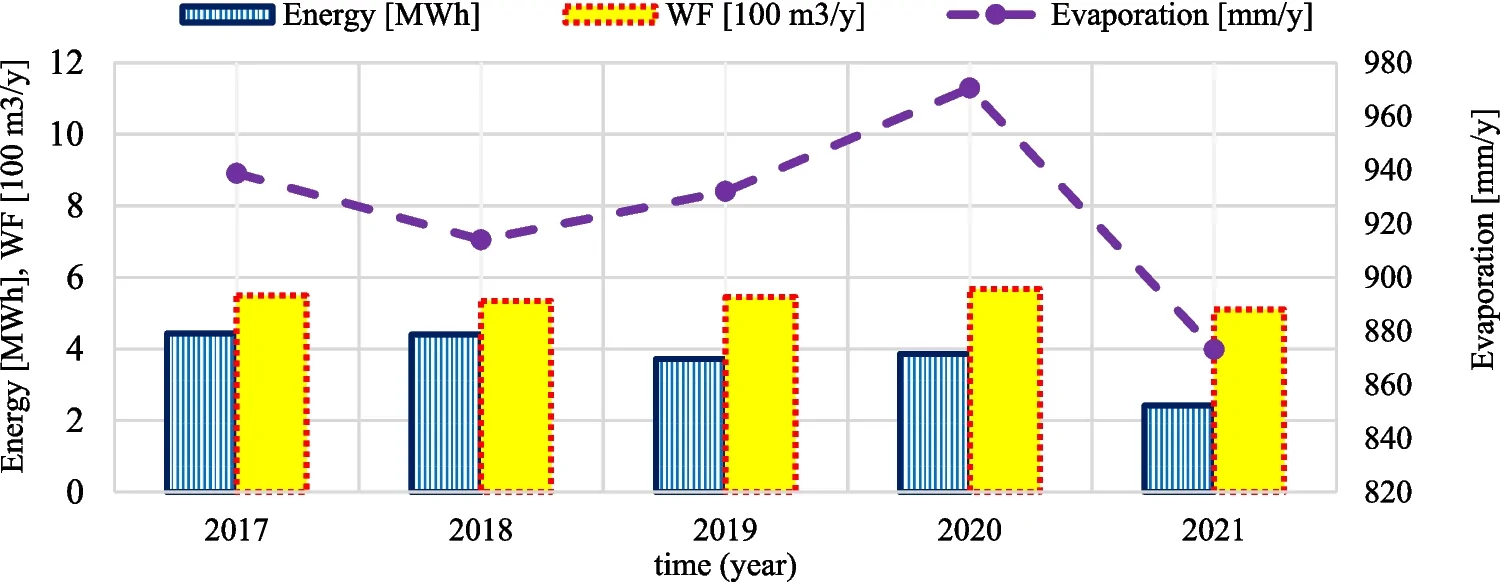
The water footprint for Dridu development

Thus, the proposed system is highly efficient in producing environmentally renewable power, and the energy ratio 683.4 results indicate that the FPV systems with small coverage area can produce higher energy than MHPP in Dridu reservoir.

In this study, an analysis of how the CC can influence the WF and CF of the shallow Dridu reservoir has been also investigated. Thus, for Dridu reservoir, currently the average value of CF is 120.23 [kg CO_2e_/MWh] and, when considering the installation of the FPV system which covers only 2 ha of the reservoir, the CF decreases by 28.13%, while the WF by 30% (Fig. 5).

**Fig. 5:**
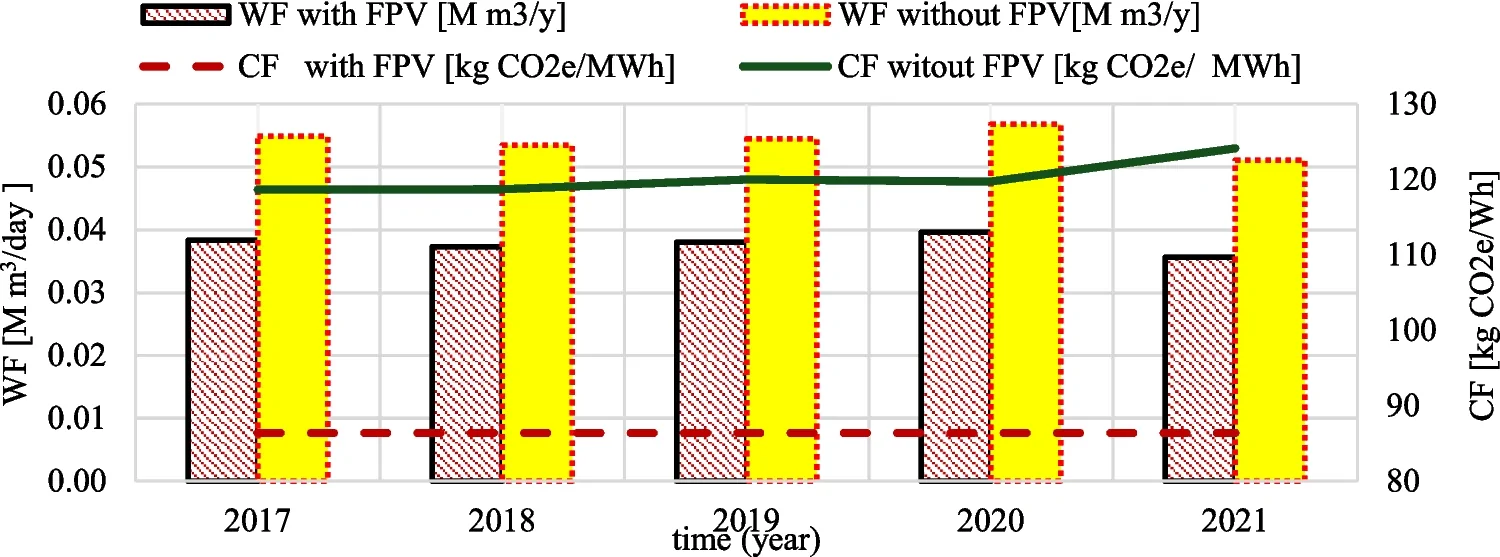
The variation of WF and CF in Dridu reservoir

In terms of CO_2_ emissions reduction, the FPV solutions proposed for Dridu reservoir generate 3.253 GWh/year of clean energy with zero GHG emissions. Thus, since the conversion factor is 230 kgCO_2e_/MWh for each 1 MWh Romania mix, the GHG emissions reduction associated with the proposed solutions will be 748.2 tCO_2e_/year. For CC scenarios, it is noted that WF will increase by 10.4% on average (Fig. 6), while the carbon footprint has no major variations (between 0.3 and 0.7%).

**Fig. 6:**
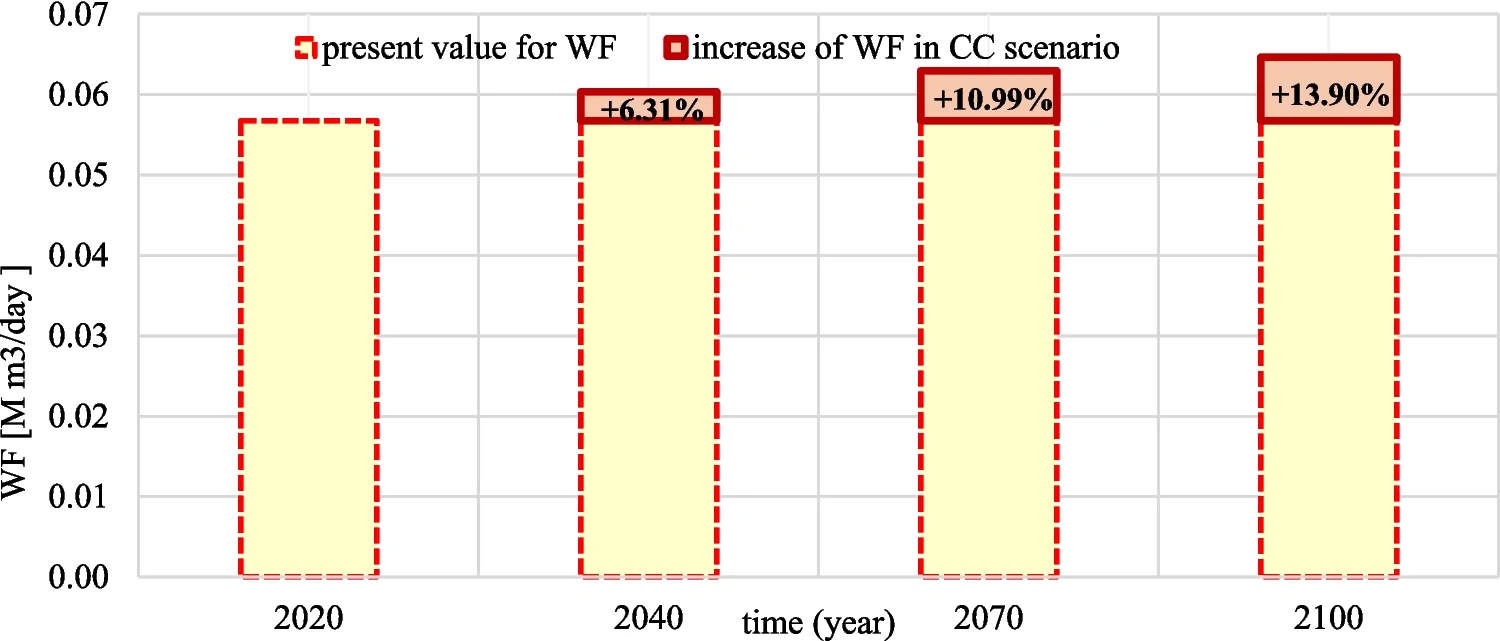
The variation of WF in CC scenario for Dridu reservoir

## Discussion

As seen from the results, the installation of a 3 MW FPV plant at Dridu reservoir will result in an annual energy of 3.253 GWh/y, with an energy density of 162.65 GWh/km^2^, and requires a 0.2 km^2^ surface. The annual electricity generation potential of FPV resulted in 1.08 GWh/MW, which is comparable with other values reported: 1.032 up to 1.29 (Kichou et al. 2022).

The system can provide clean energy with maximum values in June and July, of around 430 and 450 MWh/year respectively, and minimum values during December and January of 90 and 110 MWh/year, in good agreement with other studies for similar reservoirs (Hayibo and Pearce 2022).

The WF for the Dridu MHPP, computed for the 5 years analyzed, led to an average value of 0.054 10^6^ m^3^ and a value of 0.089 10^6^ m^3^/year for water savings was obtained. These data are well correlated with the values reported by Hayibo in 2022 or Razeghi-Jahromi in 2024 (Hayibo and Pearce 2022; Razeghi-Jahromi et al. 2024). If this saved water is used to generate electricity, the reservoir’s MHPP will produce an additional 0.45 MWh each day.

Following the study conducted in 2023, Liu et al. concluded that the effects of FPV in terms of energy generation, as well as the quantity and quality of water resources, can mitigate the impact of global climate change. They concluded that FPV systems can mitigate the increase in water temperature caused by climate change, so that covering 8.12% of the lake’s surface with FPV reduces the water temperature by approximately 0.3 °C (Liu et al. 2023). At the same time, the construction of FPV significantly reduces the concentration of dissolved oxygen (DO) in the water, which affects aquatic organisms, especially fish populations (Hardenbicker et al. 2017). There are studies that show that FPV indirectly affects the concentrations of DO and nutrients in the water body, in the sense of mitigating the stratification phenomenon and may help reduce the release of nutrients from sediments caused by anoxic conditions at the bottom (Fanga and Stefan 2009).

The most relevant possible impacts of climate change in Romania are changes in temperature and precipitation, along with more frequent extreme weather events (storms, floods, and droughts), and adapting water resources to these events is a concern both at the national and European levels. Adapting aquatic ecosystems to the projected climate changes, whether we are talking about rising temperatures or prolonged drought periods, also requires stricter management of water volumes in HP reservoirs, whether we consider methods to reduce evaporation or think about improving their trophic level.

The present study analyzes how the increase in temperatures caused by CC could be reflected in the WF and CF of the shallow Dridu reservoir, which has complex use. GHG emissions from reservoir construction and operation vary based on the type, size, and location, and for Dridu reservoir, the present-day mean value for CF is 120.23 [kg CO_2e_/MWh]. FPV can help prevent CC impacts both through reduced carbon emissions from increased global energy consumption and through water resource management, given the global dilemma of water scarcity (Exley et al. 2021). Consequently, by covering 2 ha of the reservoir surface with FPV, and in this way modifying the sun exposure and wind action, the biogeochemical pathways are affected, thus the production and release of CO₂ and CH₄ into the atmosphere, and both WF and CF decrease on average by 30% for WF and 28.13% for CF.

Ma et al. in 2022 noted that 1 MW FPV could reduce CO_2_ emissions by about 981 tons per year compared to coal-fired power generation in China (Ma et al. 2022). Given the fact that, according to Nowtricity (www.nowtricity.com/country/romania), the conversion factor is 230 kgCO_2e_/MWh for each 1 MWh Romania mix, the GHG emissions reduction associated with the FPV system on Dridu reservoir will be 748.2 tCO_2e_/y.

An increase of over 10% of the WF has been obtained for the CC scenarios, which is expected considering the increase of temperature forecasted for the study area, but no major changes have been found for CF.

## Conclusions and policy formulation

Floating solar technology, which combines efficiency and ecological sensitivity, is quickly gaining popularity not just for its energy-generating capabilities but also for its minimal environmental impact.

Romania committed to a 78% reduction of GHG by 2030; thus, green and clean energy projects are compulsory, and several policies regulate the production of electricity from renewable sources. However, dedicated support schemes and infrastructure development for electricity are still needed. At the same time, the report on the State of the Climate, 2024, emphasizes the need for developing resilience to CC, based on the recorded alterations in climate at national scale. Given the increasing temperature and the reported decrease in streamflow in the southern and southeastern areas of the country, better understanding of the CC impacts is needed, followed by specific solutions for mitigation of these effects. Another important aspect is related to the rise of the awareness regarding the CC impact and the measures which can be used to limit the negative effects. These could be included in a regulation, which is currently missing in Romania, even if there has been a proposal for a CC law for several years.

Since 2024, Romania has permitted the deployment of FPV systems, limiting the covering of the reservoir surface to a maximum of 20%. In this context, in the present study, we aimed to highlight the changes that occur with the modification of the reservoir water temperature as an effect of CC on the WF and CF and to see if the implementation of FPV on Lake Dridu can constitute an adaptation solution for this water resource to climate change. This study makes an in-depth and comprehensive investigation of the evolution of WF and CF for Dridu development. We found that the capacity installed per FPV footprint unit of area is 105 W/m^2^ and that this is in good standing with values reported in other studies. Using the Penman–Monteith method, the resulting water savings potential is estimated at 0.089 10^6^ m^3^/year, with 0.2% of the lake surface covered with FPV. Furthermore, it examines the impact of the installation of a 3 MW floating photovoltaic park on the two footprints, including the climate change predictions for the studied area. Related to the influence of FPV on WF and CF for Dridu reservoir, it is expected to decrease, representing a viable solution to combat the effects of climate change in the water mass.

Summarizing the research carried out, this paper militates for the consideration of FPV systems as a clean and sustainable alternative in energy production, but also in increasing the resilience and sustainability of water resources in the context of climate change. The innovative approach results from the connection of FPV, WF, CF, and CC. The limitations of this study are related to the assessment of WF and CF based on estimations, not on measured data, as well as from the fact that it does not consider the potential impact of FPV on water quality, the ecosystem functions, and the results are theoretical.

Nonetheless, different stakeholders can use the presented methodology to identify the most appropriate strategy for conserving the water quantity and producing clean energy.

In the authors’ opinion, a good idea for Romanian authorities is to permit and even finance the deployment and operation of FPV systems on small reservoirs, which can be used for intensive studies regarding the efficiency of different FPV systems (different arrangements and mooring systems, FPV energy output), as well as the impacts they have on water resources (e.g., water quality indicators, GHG emission, water savings).

Future research should focus on on-site testing of FPV projects to clarify the environmental impact, but also on systematic monitoring programs for both the quality indicators of the water as well as the CO_2_ emissions generated by the water body. In particular, for Dridu reservoir, a very vulnerable water body to eutrophication phenomenon, the future studies will aim to establish which are the benefits of such an FPV system on the trophic state.

## Data Availability

The datasets used and obtained during the current study are available from the corresponding author on reasonable request.

## Abbreviations

*A*: Area of the reservoir
AE: Area-to-electricity ratio
AGE: Reservoir age of functioning
CC: Climate change
CF: Carbon footprint
CO_2_: Carbon dioxide
CH_4_: Methane
E: Evaporation
FPV: Floating photovoltaic
GHI: Annual global horizontal irradiation
GHG: Greenhouse gas
GW: Gigawatt
HPP: Hydroelectric power plant
MHPP: Micro hydroelectric power plant
M m^3^: Million m^3^
MW: Mega-watt
POA: Plane-of-array irradiation
PR: Performance ratio
PV: Photovoltaic
RE: Renewable energy
SDG: Sustainable Development Goal
WF: Water footprint
